# The Impact of Aortic Arch Morphology on Periprocedural Stroke in Transcatheter Aortic Valve Replacement

**DOI:** 10.3390/jcm14041045

**Published:** 2025-02-07

**Authors:** Stephanie Voss, Katerina Rusa, Caterina Campanella, Teodora Georgescu, Keti Vitanova, Hendrik Ruge, Andrea Amabile, Konstantinos Sideris, Markus Krane, Melchior Burri

**Affiliations:** 1 Department of Cardiovascular Surgery, Institute Insure, German Heart Centre Munich, School of Medicine & Health, Technical University of Munich, Lazarettstrasse 36, 80636 Munich, Germanycampanella@dhm.mhn.de (C.C.); georgescu@dhm.mhn.de (T.G.); vitanova@dhm.mhn.de (K.V.); ruge@dhm.mhn.de (H.R.); or amabilea@upmc.edu (A.A.); sideris@dhm.mhn.de (K.S.); burri@dhm.mhn.de (M.B.); 2 Division of Cardiac Surgery, Department of Cardiothoracic Surgery, University of Pittsburgh, Pittsburgh, PA 15213, USA; 3 UPMC Heart and Vascular Institute, University of Pittsburgh Medical Center, Pittsburgh, PA 15219, USA; 4 DZHK (German Centre for Cardiovascular Research)—Partner Site Munich Heart Alliance, 81377 Munich, Germany

**Keywords:** TAVR, aortic arch, ischemic stroke, aortic arch morphology

## Abstract

**Objectives**: Stroke after transcatheter aortic valve replacement (TAVR) continues to be one of the most concerning complications. Anatomical variations of the aortic arch may increase the risk of embolic debris entering the brain during transfemoral catheter manipulation. We aimed to analyze the influence of aortic arch morphology on the occurrence of ischemic stroke during TAVR. **Methods**: We performed a retrospective, 1:2 propensity-matched case–control study to compare patients with and without periprocedural stroke (defined according to the Valve Academic Research Consortium III endpoints) after transfemoral TAVR between June 2007 and September 2022. Multi-slice computed tomography (MSCT) analysis of pre-TAVR aortograms was performed to investigate arch anatomy, configuration, curvature, and the take-off angles of the epi-aortic vessels. **Results**: A total of 2371 patients were enrolled in this study. Periprocedural ischemic stroke was observed in 67 patients. After propensity score matching, this study included 201 patients: 67 (case) vs. 134 (control). The mean age was 80.0 ± 13.2 and 81.6 ± 6.4 years (*p* = 0.5), and the mean Euroscore II was 4.1 ± 3.6 and 4.3 ± 41 (*p* = 0.7). There was no difference in arch anatomy (*p* = 0.2) and configuration (*p* = 0.8) between the groups. Arch curvature (*p* = 0.9) and angulation of the brachiocephalic (*p* = 0.3) and left common carotid artery (*p* = 0.058) also did not differ significantly between the case and control groups. **Conclusions**: MSCT analysis in this propensity score-matched study found no correlation between aortic arch geometry and TAVR-associated stroke.

## 1. Introduction

Periprocedural stroke after transcatheter aortic valve replacement (TAVR) occurs in up to 2.3–4.3% [1,2,3] of patients and continues to be one of the most devastating complications. Cerebral emboli are the primary cause of most TAVR-associated intraprocedural strokes [4]. Transfemoral access requires retrograde insertion of large devices and accessory guidewires, which mechanically interact with the aortic arch vessel wall, potentially provoking intraprocedural dislodgment of debris [5,6,7]. Catheter navigation in special geometrical variations such as a steep configuration of the aortic arch might increase the contact forces with the aortic vessel wall [8], potentially increasing the risk of cerebral embolization, as previously described in thoracic endovascular aortic repair studies [9,10]. Another important issue, potentially influencing embolic stroke manifestation during THV steering and positioning, might be the anatomical orientation of the epi-aortic vessels originating from the arch. Fluid dynamic simulations in aortic arch models have indeed found that atypical configurations of the epi-aortic arteries, such as the bovine arch anomaly, might play a substantial role in embolus transport and stroke propensity [11,12,13]. However, studies investigating TAVR-associated strokes in relation to aortic arch geometry are missing. Currently, the prevention of periprocedural stroke has especially become an important therapeutic goal with the development of dedicated cerebral embolic protection devices [14,15,16]. Should evidence confirm that aortic arch morphology influences the incidence of embolic stroke, the targeted use of cerebral embolic protection devices might be warranted in this specific subset of TAVR patients. Thus, we aimed to evaluate the influence of aortic arch anatomy on the occurrence of ischemic stroke during TAVR using multi-slice computed tomography (MSCT) analysis in a propensity score matched case–control study.

## 2. Materials and Methods

### 2.1. Patients

We conducted a retrospective, propensity score matched, case–control analysis of all patients who received a TAVR procedure at the Department of Cardiovascular Surgery at the German Heart Center Munich between June 2007 and September 2022. Patients with non-femoral TAVR access, conversion to surgical aortic valve replacement, usage of a cerebral embolic protection device, significant carotid stenosis (>70%), and missing data points were excluded from this study. The case group included all patients with a TAVR-associated periprocedural (≤30 days) overt ischemic stroke fulfilling the Neurologic Academic Research Consortium Type I (NeuroARC) [17] criteria based on the Valve Academic Research Consortium (VARC-III) endpoint definitions [18]. Patients with missing neuroimaging datasets, onset of neurological signs without neuroimaging evidence of central nervous system (CNS) infarction, need for intraoperative resuscitation, and other not ischemic related periprocedural causes of stroke (procedure-related intraoperative aortic dissection, hemorrhagic stroke, covert CNS infarction) were further excluded from the case group. After identifying the case group, a 1:2 propensity matching was conducted to find patients with similar clinical and epidemiological characteristics among those who underwent TAVR without experiencing periprocedural neurological events (≤30 days). These patients served as the control group. This study adhered to the Declaration of Helsinki and received approval from the local ethics committee of the Technical University of Munich (approval reference number: 2023-56-S-NP).

### 2.2. Stroke Definition

All strokes included in this study trial met the NeuroARC Type I criteria (Valve Academic Research Consortium III) for ischemic, symptomatic, periprocedural stroke [18]. A symptomatic ischemic stroke, as defined by the Valve Academic Research Consortium (VARC-III) endpoint definitions, was characterized by the acute onset of focal neurological symptoms, accompanied by neuroimaging evidence of central nervous system infarction in the relevant brain territory [18]. All neuroimaging reports, including brain computer tomography scans and brain magnetic resonance imaging, were reviewed and compared with the corresponding clinical data. Periprocedural stroke was defined as a symptomatic, ischemic stroke that occurred either during the index hospitalization or within 30 days following TAVR [18].

### 2.3. Aortic Arch MSCT Measurements and Definitions

All MSCT scans made as part of the preoperative TAVR planning were analyzed using the automated software program 3mensio^TM^ Structural Heart (version 10.2, Pie Medical Imaging, Maastricht, The Netherlands). A comprehensive description of the imaging analysis protocol for the respective CT measurements has been published previously by the authors [19,20,21]. The following imaging parameters were defined prior to evaluation and subsequently analyzed by two independent cardiac surgeons with experience in cardiovascular imaging.

**Figure 1:**
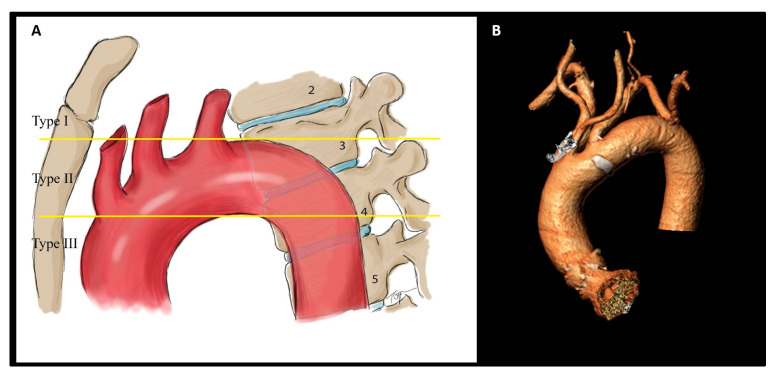
Calculation of aortic arch configuration type based on the origin of the supra-aortic arteries (**A**). Three-dimensional MSCT reconstruction of a Type III steep aortic arch (**B**).

1. Anatomical variations of the aortic arch
  - Normal aortic arch with a separated origin of the brachiocephalic, left common carotid, and left subclavian artery.
  - Bovine arch Type I presenting with a common origin for the brachiocephalic and left common carotid artery.
  - Bovine arch Type II with a separate origin of the left common carotid artery from the brachiocephalic artery.

2. Configuration of the aortic arch according to the origin of the supra-aortic arteries
  - Aortic arch Type I with all supra-aortic branches originating at the level of the outer aortic curvature.
  - Aortic arch Type II with at least one of the supra-aortic branches originating between the level of the outer and inner aortic curvature.
  - Aortic arch Type III with at least one of the supra-aortic branches originating under the level of the inner aortic curvature (Figure 1) [21,22].

3. Aortic arch curvature

**Figure 2:**
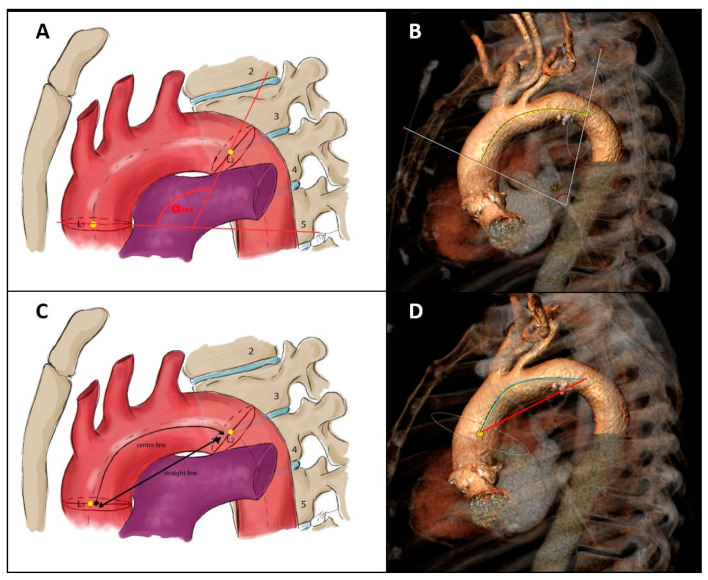
Assessment of aortic arch curvature. Schematic illustration of calculating the maximum aortic arch angulation (**A**) and aortic arch tortuosity index (**C**) relative to predefined landmarks (L1 = reference point at the level of the bifurcation of the pulmonary trunk; L2 = reference point at the level of the fourth thoracic vertebra body upper edge). MSCT-based measurements of the corresponding values (**B**,**D**).

Assessment of aortic arch curvature included the calculation of the maximal aortic arch angulation (AAA) as well as the determination of the aortic arch tortuosity index (TI) following the description by Boufi et al. [6]. For evaluating the AAA, reference points along the aortic centerline at the pulmonary trunk bifurcation and the fourth thoracic vertebra were used. The angle between perpendicular planes at these points defined the aortic arch angulation (Figure 2A,B). The aortic arch TI was calculated as follows: [(center-line distance)/(straight-line distance) − 1] × 100 [6,19]. The centerline distance was defined as the true length along the 3D centerline between the previously selected landmarks, whereas the straight-line distance represents the linear distance between these two points (Figure 2C,D).

4. Aortic arch take-off angles

The following angles, as described previously by the authors, were assessed [19,22]:

- AA/BA angle = angle between the aortic arch and the brachiocephalic artery.
- AA/LCC angle = angle between the aortic arch and the left common carotid artery.

### 2.4. Clinical Data Collection

Baseline demographics, procedural characteristics, and postoperative data were prospectively recorded in our dedicated TAVR database and updated to the latest VARC-III recommendations [18].

### 2.5. Statistical Analysis

After checking for normal distribution, data were provided as count, mean ± standard deviation, or median with interquartile range as appropriate. Groups were compared using unpaired *t*-test, Wilcox rank sum test, and Fisher’s exact test as appropriate. The matching was performed using a combination of exact matching (valve type) and propensity score matching (including age, gender, history of atrial fibrillation, Euroscore II, preoperative stroke, and previous operation). A logistic regression model was used to calculate the propensity score, and the nearest neighbor matching protocol was employed for the matching process. To ensure data completeness for matching, the number of variables was limited to only those deemed clinically relevant. The level of inter-observer and intra-observer consensus for all study-specific aortic arch imaging measurements was tested by calculating the inter- and intra-class correlation coefficient (ICC), as previously described [19,21,23]. ICC values were categorized as follows:

- ≤0.5: poor reliability;
- 0.5–0.75: moderate reliability;
- 0.75–0.9: good reliability;
- ≥0.9: excellent reliability.

A *p*-value of less than 0.05 was considered statistically significant. Statistical analyses were performed by using the R environment version 4.2.1 [24] and BM SPSS Statistics 22.0 software (IBM Corp, Armonk, NY USA).

## 3. Results

### 3.1. Patients and Matching Results

**Figure 3:**
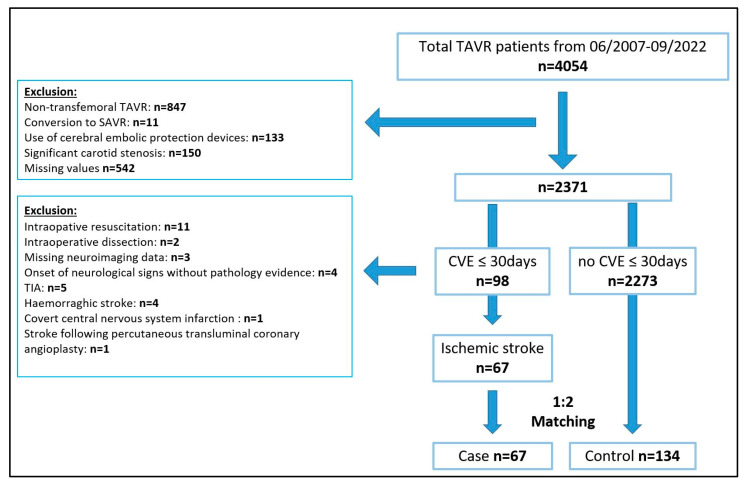
Flow diagram of study population.

The study flow chart is displayed in Figure 3. Of 4054 patients undergoing TAVR at our institution, 2371 qualified for the study based on the inclusion/exclusion criteria. Of those, 98 patients (4.1%) had a periprocedural cerebrovascular event (≤30 days); 67 of those met the criteria for inclusion into the case group.

The 1:2 matching resulted in a control group of 134 patients without periprocedural cerebrovascular events. The distribution of the variables included in the matching is shown in Table 1. Prior to matching, there was a significant difference in the valve type used between the case and the control groups. Most notably, there was a higher rate of first-generation self-expanding valves in the case group (28% vs. 10%), whereas the rate of balloon-expandable valves was higher in the control group. Furthermore, patients without periprocedural stroke had a slightly higher Euroscore II (5.0 ± 6.2 vs. 4.1 ± 3.6) and a higher rate of previous cardiac operations (14.2% vs. 6.0%). After applying the matching procedure, the 134 matched patients, who represent the control group, showed perfect agreement concerning the implanted valve type and were similar in terms of the remaining variables used for matching (Table 1).

### 3.2. Baseline and Perioperative Patient Characteristics

Baseline characteristics not included in the matching protocol were comparable between the case and the control group (Table 2): median age was 83 years [IQR 65–93] and 82 years [IQR 53–94] (*p* = 0.7), and the previous stroke rate was 13.4% and 10.4% (*p* = 0.6) in the case and control group, respectively. The rate of balloon-expandable valves was 34.3% in both groups, with almost identical rates of pre- (59.7% vs. 58.2%, *p* = 0.9) and post-dilatation (32.8% vs. 33.6%, *p* = 1.0).

### 3.3. MSCT Measurements

**Figure 4:**
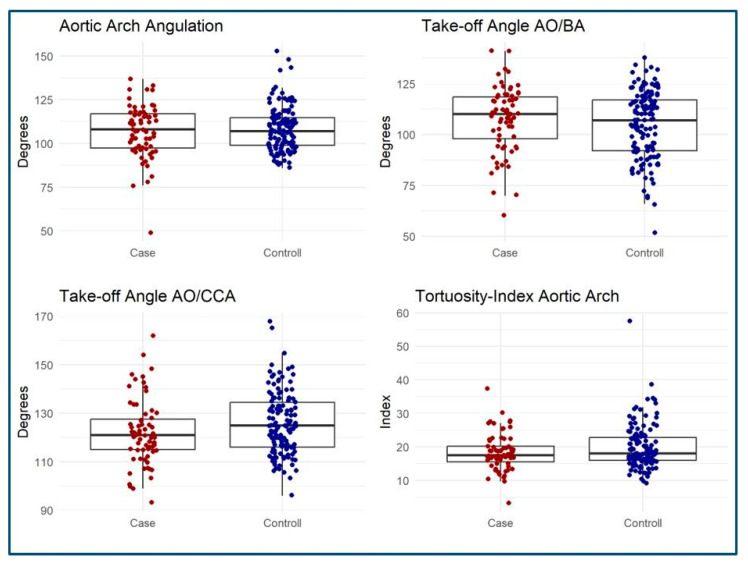
Boxplots illustrating MSCT measurements in the case and control groups.

Retrospective aortic arch morphology MSCT evaluation was performed in all patients (Table 3). The measurements showed good to excellent reliability in terms of inter- and intra-observer differences (Tables S1 and S2, Supplementary Materials). There was no difference regarding the anatomy of the aortic arch (*p* = 0.2) with a standard aortic arch anatomy being the predominant morphology in both groups (case group: 74%, control group: 66.4%). Bovine arch anatomy was found in 25.3% of patients with stroke and in 33.5% of patients without. Aortic arch configuration did not differ either among groups (*p* = 0.8) (Table 3). A Type II configurated aortic arch was the most common pattern with 71.6% in both groups (*p* = 0.8). There was also no difference in aortic arch angulation (*p* = 0.9) and tortuosity index (*p* = 0.3) (Table 3) (Figure 4). The determination of the AO/BA angle was also similar in both groups (median 111° vs. 107°, *p* = 0.3), as well as the AO/CCA angle (121° vs. 125°, *p* = 0.058) (Figure 4).

## 4. Discussion

Despite advances in technology and expertise, which have lowered the incidence of stroke post-TAVR over time, this complication remains a serious event that significantly affects both survival and quality of life [1]. Recent research has focused on the detection of risk factors for periprocedural stroke, including patient characteristics and procedural factors such as THV selection, access route, and aortic valve anatomy. However, the anatomical morphology of the aortic arch as a predictor for TAVR-associated stroke has not yet been systematically investigated. This study is the first of its kind to analyze how aortic arch anatomy, configuration, angulation, and the take-off angles of the epi-aortic arteries impact TAVR-associated stroke in a propensity-matched case–control study. However, imaging examinations in our patients showed no association between TAVR-associated stroke and aortic arch morphology.

We initially hypothesized that the presence of a bovine arch anatomy might serve as a predictive feature for embolic stroke in TAVR patients. This atypical supra-aortic branching pattern, occurring in up to 31% of individuals [21,25], constitutes the most common anatomical arch variation and has been linked to a higher stroke incidence in the general population [13,26,27]. Previous biomechanical studies indicated that such atypical branching patterns of the supra-aortic vessels may impact flow hemodynamics and embolus transport [11,12,13]. The shared origin of the brachiocephalic and the left common carotid arteries also creates a wider, single pathway for potential embolic debris, which might increase the stroke risk in patients with this anatomical variation [28]. However, our propensity-matched case–control study could not confirm this clinically. The frequency of bovine arch anatomy was 25.4% versus 33.5% among TAVR patients with and without a periprocedural stroke, respectively (*p* = 0.2).

Besides our investigation, there is only one recently published registry study by Russo et al., which also addresses the scarcely explored topic of aortic arch morphology in relation to stroke rates in the TAVR population [28]. However, Russo and colleagues present divergent study findings and report that a bovine arch is associated with a twofold increase in TAVR-associated stroke among TAVI patients with a bovine arch (n = 495) compared to patients with a non-bovine anatomy (n = 2280) [28]. There are several reasons why the results of Russo and colleagues could not be reproduced in our study. First, Russo et al. did not limit their cohort to patients with transfemoral access and did not exclude patients requiring conversion to SAVR or other TAVR-related complications such as aortic dissection. Second, Russo`s study also included patients with hemorrhagic infarctions as well as those with neurological symptoms without neuroimaging evidence of central nervous system infarction. In contrast, this patient population was intentionally excluded from our analysis. Our aim was to focus on a more specific patient cohort presenting with symptomatic, ischemic stroke to ensure the precision of our findings. Third, the method of correction for possible confounders was chosen differently in the two studies. While Russo et al. used a multivariate regression model to calculate the isolated effect of the bovine arch anatomy, we performed propensity score matching. Due to the study design, Russo and colleagues analyzed a significantly larger population. Nevertheless, the reliability of the two statistics largely depends on the number of patients with periprocedural stroke, which was similar in both studies. Fourth, an important confounder might be the usage of different transcatheter heart valve systems, as distinct implantation techniques might be linked to varying incidences of periprocedural stroke rates [14,29]. The analysis of our unmatched cohorts revealed a significant difference in valve types between the stroke and control groups (*p* = < 0.001), which was balanced after matching (*p* = 1.0). Russo et al. did not correct for valve type in their logistic regression model [28]. Furthermore, the study investigators did not provide a clear description of their CT evaluation protocol. In contrast, the MSCT evaluation in our study follows a comprehensive and highly standardized protocol, detailing each individual measurement and specifying the exact software used.

Besides bovine arch anatomy, we assumed that aortic arch steepness and the corresponding take-off angles of the supra-aortic arteries might be further potential determinants for procedure-related strokes. In our study, arch steepness was assessed by the evaluation of aortic arch configuration type and the measurement of arch curvature. Unlike bovine arch anatomy, there are currently no data concerning the distribution of arch steepness in general and TAVR populations and its potential influence on stroke rates. Navigating the THV catheter in a steeply configured arch might increase the contact forces on the vessel wall, potentially resulting in a higher risk of cerebral embolization [8]. Neurointerventional studies have found that a Type III arch could be associated with prolonged catheter manipulation time and therefore increased periprocedural risk [30]. In our study cohort, the Type II arch emerged as the most common configuration, with similar arch angulations and take-off angles of the supra-aortic arteries in both groups. Thus, arch steepness and supra-aortic take-off angles could also not be identified as predictors for periprocedural stroke.

Although our investigation did not demonstrate any significant impact of aortic arch anatomy on the incidence of TAVR-associated stroke, important clinical insights can still be drawn. Based on our findings, we do not consider it necessary to use additional cerebral embolic protection systems in patients with atypical aortic arch morphologies, such as a bovine arch configuration. This conclusion is particularly relevant given that current cerebral protection devices have not shown benefits regarding hard clinical endpoints [31]. Additionally, studies showed that the usage of dual-filter-based cerebral embolic protection devices in TAVR patients presenting with bovine arch anomalies has been associated with higher rates of device implantation failure, owing to the more complex navigation maneuvers required [19,32]. According to our MSCT-based propensity-matched analysis, we would not take any special precautions in this patient population.

Our study presents findings from a retrospective, single-center investigation focusing on the correlation between stroke incidence and aortic arch morphology in TAVR patients. Therefore, stroke severity was not evaluated. Although we performed propensity score matching to minimize confounders while isolating the effect of aortic arch morphology, procedural factors may still be confounded by advancements in technique, operator experience, and patient selection over time. Additionally, factors such as the amount of aortic calcification and periprocedural data, including the use of specific medications, may have influenced the outcomes.

## 5. Conclusions

MSCT analysis in this propensity-matched case–control study found no association between aortic arch morphology and TAVR-associated stroke. Therefore, it is not possible to elaborate on the risk of periprocedural stroke solely based on the morphology of the aortic arch.

## Figures and Tables

**Table 1:** Balancing of matched variables before and after matching.

	Prematch				Postmatch			
	Case	Control	SMD	*p*-Value	Case	Control	SMD	*p*-Value
	n = 67	n = 2273			n = 67	n = 134		
Female gender	35 (52.2%)	1089 (47.9%)	0.087	0.5	35 (52.2%)	70 (52.2%)	0	1.0
Atrial fibrilation	16 (23.9%)	640 (28.2%)	0.095	0.5	16 (23.9%)	26 (19.4%)	0.11	0.5
Preoperative stroke	9 (13.4%)	241 (10.6%)	0.092	0.4	9 (13.4%)	14 (10.4%)	0.094	0.6
Previous operation	4 (6.0%)	323 (14.2%)	0.238	0.07	4 (6.0%)	8 (6.0%)	0	1.0
Age (Y)	80.0 ± 13.2	79.7 ± 8.9	0.042	0.8	80.0 ± 13.2	81.2 ± 6.4	0.128	0.5
Euroscore 2	4.1 ± 3.6	5.0 ± 6.2	0.145	0.058	4.1 ± 3.6	4.3 ± 4.1	0.055	0.7
Valve type				<0.001				1.0
Medtronic CoreValve (Metdronic, Minneapolis, MN, USA)	19 (28.4%)	229 (10.1%)			19 (28.4%)	38 (28.4%)		
Medtronic Evolut R (Metdronic, Minneapolis, MN, USA)	14 (20.9%)	561 (24.7%)			14 (20.9%)	28 (20.9%)		
Edwards Sapien Ultra (Edwards Lifesciences, Irvine, CA, USA)	12 (17.9%)	527 (23.2%)			12 (17.9%)	24 (17.9%)		
Edwards Sapien 3 (Edwards Lifesciences, Irvine, CA, USA)	10 (14.9%)	523 (23.0%)			10 (14.9%)	20 (14.9%)		
Medtronic Evolut Pro (Metdronic, Minneapolis, MN, USA)	5 (7.5%)	90 (4.0%)			5 (7.5%)	10 (7.5%)		
Boston Scientific Lotus (Boston Scientific, Watertown, MA, USA)	4 (6.0%)	134 (5.9%)			4 (6.0%)	8 (6.0%)		
Symetis Acurate Neo (Boston Scientific, Watertown, MA, USA)	2 (3.0%)	80 (3.5%)			2 (3.0%)	4 (3.0%)		
Edwards Sapien XT (Edwards Lifesciences, Irvine, CA, USA)	1 (1.5%)	24 (1.1%)			1 (1.5%)	2 (1.5%)		
Other	0 (0.0%)	105 (4.6%)			0 (0.0%)	0 (0.0%)		

Notes: Groups were compared using Fisher’s exact test, chi-square, or *t*-test. SMD: standardized mean difference.

**Table 2:** Baseline and perioperative patient characteristics.

Characteristics	Case	Control	*p*-Value
n	67	134	
Age (y)	83 [65–93]	82 [53–94]	0.7
Female gender (n)	35 (52.2%)	70 (52.2%)	1.0
Body mass index (kg/m^2^)	25.7 [16.5–46.8]	27.0 [3.3–45.7]	0.09
Coronary artery disease (n)	37 (55.2%)	70 (52.2%)	0.8
Hypertension (n)	59 (88.1%)	123 (91.8%)	0.4
Diabetes (n)	18 (26.9%)	43 (32.1%)	0.5
Peripheral artery disease (n)	11 (16.4%)	17 (12.7%)	0.5
Previous cardiac procedure (n)	4 (6.0%)	8 (6.0%)	1.0
Previous stroke (n)	9 (13.4%)	14 (10.4%)	0.6
COPD (n)	11 (16.4%)	13 (9.7%)	0.2
LV-EF (n)			0.3
>50%	49 (73.1%)	88 (65.7%)	
35–50%	13 (19.4%)	34 (25.4%)	
<35%	5 (7.5%)	11 (8.2%)	
GFR (MDR) (n)	58 ± 20	65 ± 24	0.059
STS Score	4.1 ± 3.0	4.2 ± 3.3	0.8
Euroscore II	4.1 ± 3.6	4.3 ± 4.1	0.7
Year of procedure (n)			0.4
2007–2012	12 (17.9%)	16 (11.9%)	
2012–2017	24 (35.8%)	47 (35.1%)	
2017–2023	31 (46.3%)	71 (53.0%)	
Ballon-expandable valve (n)	23 (34.3%)	46 (34.3%)	1.0
Second valve implantation (n)	2 (3.0%)	1 (0.7%)	0.3
Puncture to suture (min)	73 ± 38	66 ± 39	0.2
Pre-dilatation (n)	40 (59.7%)	78 (58.2%)	0.9
Post-dillatation (n)	22 (32.8%)	45 (33.6%)	1.0

GFR: glomerular filtration rate.

**Table 3:** MSCT measurements.

MSCT Data	Case	Control	*p*-Value
n	67	134	
Anatomy			0.2
Normal	50 (74.6%)	89 (66.4%)	
Bovine I	7 (10.4%)	27 (20.1%)	
Bovine II	10 (14.9%)	18 (13.4%)	
Configuration			0.8
Type I	9 (13.4%)	15 (11.2%)	
Type II	48 (71.6%)	96 (71.6%)	
Type III	10 (14.9%)	23 (17.2%)	
AO/BA Angle (degree)	110 [60–141]	107 [52–138]	0.3
AO/CCA Angle (degree)	121 [93–162]	125 [96–168]	0.058
Arch Angulation (degree)	108 [49–137]	107 [86–153]	0.9
Tortuosity Index	17.5 [3.3–37.4]	18.1 [9.2–57.5]	0.3

## Data Availability

All relevant data generated or analyzed during this study are included in this published article.

## Supplementary Materials

The following supporting information can be downloaded at: https://www.mdpi.com/article/10.3390/jcm14041045/s1. Table S1: Intraobserver agreement for 3-dimensional multislice computed tomography measurements, Table S2: Interobserver agreement for 3-dimensional multislice computed tomography measurements.

## Abbreviations:

AAA	Aortic arch angulation
AA/BA	Angle aortic arch/brachiocephalic artery angle
AA/LCCA	Angle aortic arch/left common carotid artery angle
AF	Atrial fibrillation
CNS	Central nervous system
LVEF	Left ventricular ejection fraction
ICC	Intra-class correlation coefficient
MSCT	Multi-slice computed tomography
VARC III	Valve Academic Research Consortium III
TAVR	Transcatheter aortic valve replacement
THV	Transcatheter heart valve
TI	Tortuosity index
