# Supplementary material for: The Impact of Aortic Arch Morphology on Periprocedural Stroke in Transcatheter Aortic Valve Replacement
Source: J Clin Med. 2025 Feb 7;14(4):1045. doi: 10.3390/jcm14041045 (PMC11856225; doi:10.3390/jcm14041045)
Supplement: Supplementary file 1 [file jcm-14-01045-s001.zip › jcm-3402836-supplementary.pdf]

Supplement

Table S1: Intraobserver agreement for 3-dimensional multislice computed tomography measurements

Parameters	Intraclass Correlation Coefficient	95% Confidence Interval	
		Lower Bound	Upper Bound
Take-off angle AO/BA	0.982	0.950	0.994
Take-off angle AA/CCA	0.980	0.895	0.994
Aortic arch angulation	0.889	0.711	0.960
Aortic arch tortuosity index	0.882	0.518	0.964

Table S2: Interobserver agreement for 3-dimensional multislice computed tomography measurements

Parameters	Intraclass Correlation Coefficient	95% Confidence Interval	
		Lower Bound	Upper Bound
Take-off angle AO/BA	0.985	0.958	0.995
Take-off angle AO/CCA	0.984	0.956	0.994
Aortic arch angulation	0.921	0.778	0.972
Aortic arch tortuosity index	0.930	0.805	0.975
